# A magnetic γ-Fe_2_O_3_@PANI@TiO_2_ core–shell nanocomposite for arsenic removal *via* a coupled visible-light-induced photocatalytic oxidation–adsorption process†

**DOI:** 10.1039/d0na00171f

**Published:** 2020-03-30

**Authors:** Yuan Wang, Ping Zhang, Tian C. Zhang, Gang Xiang, Xinlong Wang, Simo Pehkonen, Shaojun Yuan

**Affiliations:** Low-carbon Technology & Chemical Reaction Engineering Lab, College of Chemical Engineering, Sichuan University Chengdu 610065 China ysj@scu.edu.cn; Civil and Environmental Engineering Department, University of Nebraska-Lincoln Omaha NE 68182-0178 USA; College of Physical Science and Technology, Sichuan University Chengdu 610064 China; Department of Environmental and Biosciences, University of Eastern Finland 70211 Kuopio Finland

## Abstract

Arsenic polluted groundwater impairs human health and poses severe threats to drinking water supplies and ecosystems. Hence, an efficient method of simultaneous oxidation of As(iii) to As(v), and removal of As(v) from water has triggered increasing attention. In this study, a magnetic γ-Fe_2_O_3_ core–shell heterojunction nanocomposite was synthesized by means of hydrothermal crystallization of TiO_2_ on the surface of the magnetic core–shell loaded with polyaniline (γ-Fe_2_O_3_@PANI@TiO_2_). As an efficient photocatalyst coupled with adsorption, γ-Fe_2_O_3_@PANI@TiO_2_ has a high light utilization and good adsorption capacity. Notably, the nanocomposite has excellent stability at various initial pH values with good reusability. Among the co-existing ions investigated, PO_4_^3−^ has the greatest competitive reaction. The photocatalytic oxidation of As(iii) on γ-Fe_2_O_3_@PANI@TiO_2_ is dominated by the synergy of several active substances, with superoxide free radicals and photogenerated holes being the major players.

## Abstract 2

A magnetic γ-Fe_2_O_3_@PANI@TiO_2_ nanocomposite acts as an efficient photocatalyst coupled with adsorption for arsenic removal. It could achieve a good capacity of As(iii) removal under visible light.

## Graphical abstract

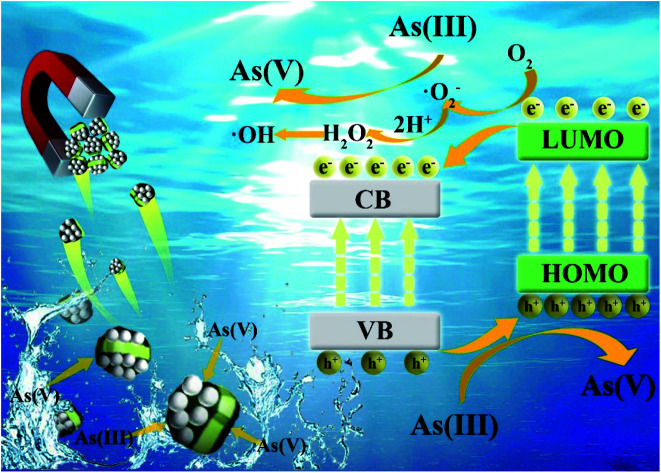

## Introduction

Arsenic is one of the many metal elements in the water environment, soil, rocks as well as in the atmosphere, and poses a serious threat due to its strong toxicity to humans and other species.^1–3^ The transformation and migration of arsenic to the biota come mainly from the water environment. Since groundwater is a main source of drinking water in many countries, its safe level in water is of vital importance to human life and ecology in general.^4,5^ Mexico, Bangladesh, India, Vietnam, China, Argentina, Chile and other 22 different countries or regions in the world are affected by arsenic pollution.^6–8^ Particularly, in Bangladesh 40 million people are reported to be at risk of arsenic poisoning.^9^ Therefore, it is imperative to develop advanced treatment systems to remove aquatic arsenic.

The common arsenic species in groundwater include As(iii) (arsenite) and As(v) (arsenate), and its specific form is affected by the redox potential (ORP) and pH.^10,11^ As(iii) has greater toxicity and mobility than As(v), leading to the poor performance of many of the removal technologies of As(iii).^12^ An efficient approach is to oxidize As(iii) to As(v) *via* a pre-treatment process as As(v) can be easily removed through adsorption, ion exchange, and coagulation.^13^ Recently, photocatalytic degradation methods have received considerable attention.^14–16^ Among them, the UV/TiO_2_ system distinguishes itself by its stability and low cost.^17–21^ However, TiO_2_ delivers only a limited adsorption capacity, resulting in an inefficient removal capacity.^22,23^ Therefore, development of systems for efficient oxidation of As(iii) to As(v) and simultaneous removal of As(v) has received increasing attention.

Yoon *et al.* used activated alumina to achieve photocatalytic oxidation and adsorption of As(iii) in both Fenton and UV/TiO_2_ systems. After four hours of reaction, the total arsenic removal efficiency could reach 90%.^23^ Miller *et al.* applied TiO_2_ impregnated chitosan beads to arsenic removal; these beads exhibited a sorption capacity of 6.4 mg g^−1^ for As(iii) under UV light but only 2.2 mg g^−1^ without UV light.^24^ Although photocatalytic oxidation coupled with adsorption for arsenic removal has triggered increasing attention, this technology still has several weaknesses, such as low light utilization efficiency, low quantum efficiency, and poor adsorption capacity. Solving the above problems relies mainly on expanding the light absorption wavelength range, increasing the light absorption rate and thereby enhancing catalyst's visible light reactivity. Thus, the current knowledge gap is how to enhance the activity and efficiency of the photocatalyst for arsenic removal by modifying the structure and composition of the catalyst.

As a conductive polymer, polyaniline (PANI) is inherently a good electron donor and a carrier of photogenerated holes.^25,26^ Meanwhile, PANI can promote the carrier migration rate, restrain the electron–hole recombination, and expand the light absorption to the visible region and improve the quantum efficiency of light utilization.^27–29^ In addition, previous studies showed that maghemite (γ-Fe_2_O_3_) has excellent adsorption properties in wastewater treatment.^30–32^ γ-Fe_2_O_3_ can efficiently adsorb As(v) onto the surface, and then be easily separated under a magnetic field.^33,34^ Therefore, we hypothesize that TiO_2_ on the surface of the γ-Fe_2_O_3_ core–shell loaded with PANI (γ-Fe_2_O_3_@PANI@TiO_2_) would significantly enhance the photocatalytic adsorption of As(iii).

In this work, for the first time we have synthesized/optimized γ-Fe_2_O_3_@PANI@TiO_2_, a bifunctional material for highly efficient removal of As(iii). This letter reports several aspects of our investigation, including the performance of the new material under the influence of initial pH, co-existing ions, initial As(iii) concentrations and the dosage of the catalyst; the removal kinetics; the reusability and the associated mechanism of the photocatalytic oxidation of As(iii).

## Experimental

### Materials and methods

#### Synthesis of γ-Fe_2_O_3_

**Fig. 1:**
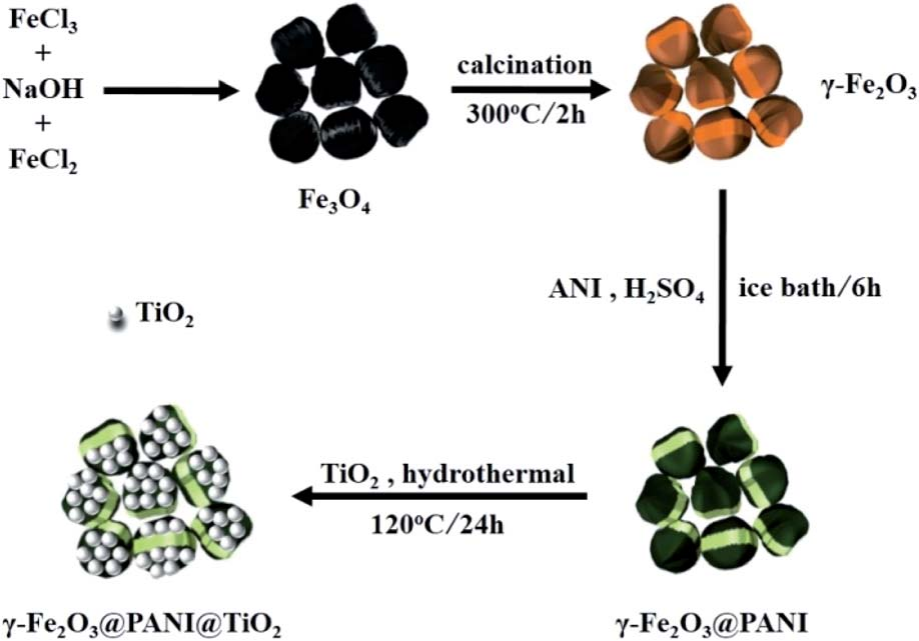
Schematic illustration of the preparation procedure of γ-Fe_2_O_3_@PANI@TiO_2_.

All chemicals were of analytical grade and used as received without further purification. 10.81 g of FeCl_3_·6H_2_O was dissolved in 40 mL of deionized water; then a 250 mL solution containing 6 g of NaOH was added to the FeCl_3_ solution under mechanical stirring, and heated to boil for 10 minutes before natural cooling; then 250 mL of 0.01 M HNO_3_ solution was used to mix the component solutions; then the precursor was collected and washed with deionized water by centrifugation until the solution pH was 7. 11.93 g of FeCl_2_·4H_2_O was dissolved in 20 mL of deionized water; then 4.2 g of NaOH was dissolved in 250 mL of deionized water. The precursor was then added to the FeCl_2_ solution, and the NaOH solution was added under mechanical stirring; then the solution was heated using an electric heating sleeve until boiling, and was kept for 30 minutes before natural cooling, and a precipitate was obtained. Then 0.01 M HNO_3_ solution was mixed with the precipitate and centrifuged until the washing solution was neutral. Finally, the cleaned product was vacuum-dried at 50 °C for 48 h, and the product was Fe_3_O_4_. γ-Fe_2_O_3_ was obtained after Fe_3_O_4_ was annealed at 300 °C for 2 h in an air atmosphere (Fig. 1).

#### Synthesis of γ-Fe_2_O_3_@PANI

0.4 g of γ-Fe_2_O_3_ was added to 80 mL of 0.5 M H_2_SO_4_ solution in an ice bath. Then, 1 g of aniline monomer was added to the above solution with mechanical stirring. Then, 4.56 g of ammonium persulfate as the initiator was added to the above solution, which then was kept in an ice bath for 6 h. Finally, the product was vacuum-dried at 50 °C for 48 h. Pure PANI was prepared under the same conditions.

#### Synthesis of γ-Fe_2_O_3_@PANI@TiO_2_

After drying, a mass of 0.2 g of γ-Fe_2_O_3_@PANI was weighed and dissolved in 120 mL of deionized water. After 30 minutes of ultrasonic water bath mixing, the γ-Fe_2_O_3_@PANI was dispersed evenly. Then, 0.8 g of TiO_2_ (P25, provided by Chengdu Kelong Chemical Reagent Co., Ltd) was added to the above solution slowly and mechanically stirred for 30 minutes at 100 RPM to dissolve and fully disperse the TiO_2_. Then, the solution was transferred to a 150 mL PPL-lined high pressure reaction vessel, which was sealed and placed in an electric drum air desiccant box, and the hydrothermal crystallization reaction was conducted at 120 °C for 24 h. After the reaction, a large amount of deionized water and anhydrous ethanol were used repeatedly and the product was centrifuged to remove unreacted TiO_2_ and other impurities. Finally, the product was freeze-dried at −50 °C for 24 h, and the obtained product is known as the γ-Fe_2_O_3_@PANI@TiO_2_ heterojunction composite material.

#### Characterization and experiments

The characterization and experiments are shown in the ESI.†

## Results and discussion

**Fig. 2:**
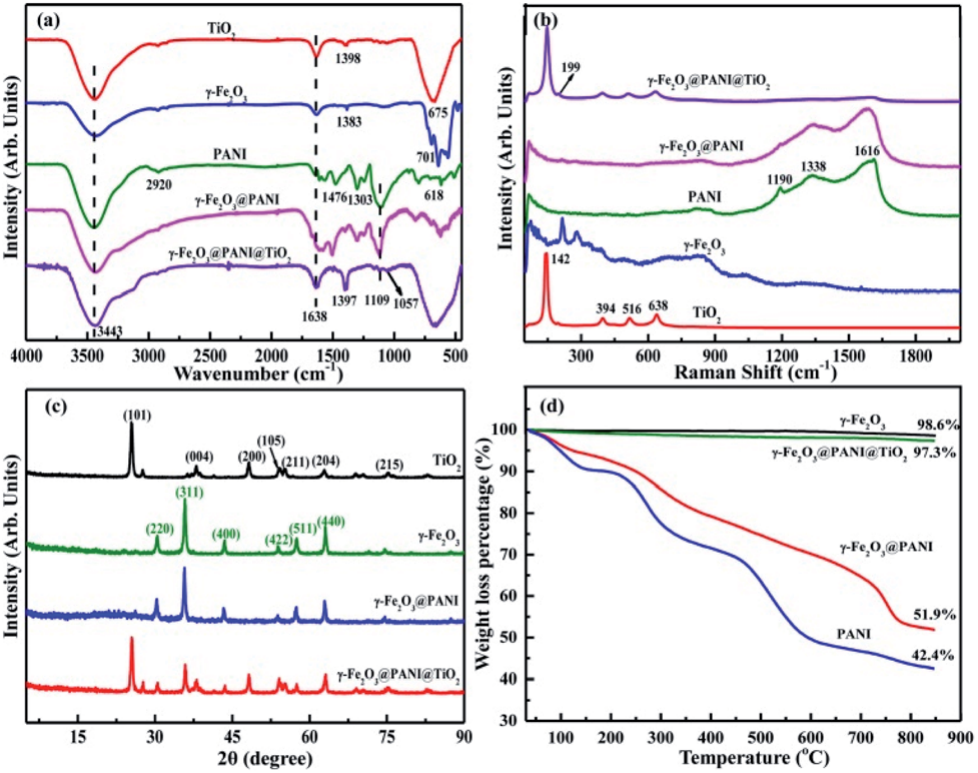
Characterization of TiO_2_, γ-Fe_2_O_3_, γ-Fe_2_O_3_@PANI, and γ-Fe_2_O_3_@PANI@TiO_2_: (a) FTIR spectra, (b) Raman spectra, (c) XRD spectra, and (d) thermogravimetric analysis (TG) curves.

Fig. S1† shows the visual appearance comparison of γ-Fe_2_O_3_@PANI@TiO_2_, γ-Fe_2_O_3_@PANI, and γ-Fe_2_O_3_. The Fourier transform infrared (FTIR) spectra are shown in Fig. 2a. The two bands at 3443 cm^−1^ and 1638 cm^−1^ are attributed to the –OH stretching vibration of surface water and the –OH bending vibration absorption of surface water molecules or carboxyl groups, respectively. The band at 675 cm^−1^ for TiO_2_ corresponds to the Ti–O–Ti stretching vibration.^35^ For γ-Fe_2_O_3_, the peak at 1383 cm^−1^ corresponds to the characteristic peak of O–H deformation, and the absorption within the range of 500–800 cm^−1^ is attributed to the Fe–O stretching vibration.^36^ After PANI coating, the spectrum of γ-Fe_2_O_3_@PANI shows the characteristic peaks of γ-Fe_2_O_3_ and PANI.^37,38^ When TiO_2_ was introduced onto the γ-Fe_2_O_3_@PANI, a new absorption peak at 1057 cm^−1^ appeared, corresponding to the C–O–Ti stretching.^39^ It is clear that the formation of Fe_2_O_3_@PANI@TiO_2_ is successful. Due to the high content of TiO_2_, the characteristic peaks of the nanocomposite and TiO_2_ are relatively consistent, while the peaks of other components are mostly suppressed. In Fig. 2b, the absorption of *E*_g_, *B*_1g_ and *A*_1g_ Raman activity characteristics of TiO_2_ at 142, 199, 394, 516, and 638 cm^−1^ is observed.^40^ After introducing TiO_2_ onto the γ-Fe_2_O_3_@PANI, the γ-Fe_2_O_3_@PANI@TiO_2_ mainly displays the characteristic peak of TiO_2_ with a weak peak of PANI, indicating the relatively high loading of TiO_2_.

The X-ray diffraction (XRD) pattern of γ-Fe_2_O_3_@PANI exhibits the (220), (311), (400), (422), (511), and (440) planes of γ-Fe_2_O_3_,^41^ and a weak broad peak in the range of 15–25° deriving from the characteristic of PANI, which indicates the successful synthesis of γ-Fe_2_O_3_@PANI (Fig. 2c). After loading TiO_2_ onto the γ-Fe_2_O_3_@PANI, all peaks of γ-Fe_2_O_3_@PANI@TiO_2_ correspond to the nature of TiO_2_ and γ-Fe_2_O_3_. The characteristic peak of PANI cannot be observed, indicating the high loading of TiO_2_, which is consistent with the result of Raman spectra. As shown in Fig. 2d, γ-Fe_2_O_3_ has less weight loss, indicating the good thermal stability. For γ-Fe_2_O_3_@PANI, the decomposition temperature in the range of 180–600 °C mainly corresponds to the weight loss of the PANI oligomer, doping agent, and the branched polymer chain. A rapid weight loss is observed at a temperature of 730 °C, which is due to the decomposition of the outer structure of PANI. In the range of 730–850 °C, the rate of weight loss is 10%, indicating the pyrolyzation of benzene and quinone ring structures. Notably, Fe_2_O_3_@PANI@TiO_2_ exhibits a good thermal stability, which is due to the introduction of TiO_2_ onto γ-Fe_2_O_3_@PANI.

**Fig. 3:**
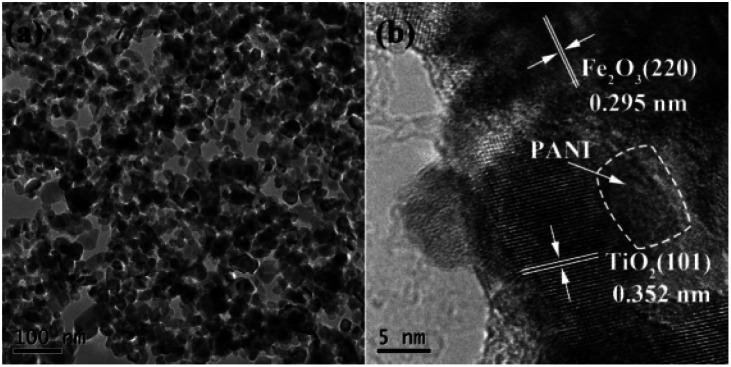
TEM images of the surface morphology of γ-Fe_2_O_3_@PANI@TiO_2_ nanocomposites at (a) low magnification and (b) high magnification.

The composite material prepared by the hydrothermal crystallization has a good dispersion (Fig. 3a). The high magnification transmission electron microscopy (TEM) image (Fig. 3b) shows a few lattice planes with a *d*-spacing of 0.352 nm, corresponding to the (101) plane of TiO_2_, and of 0.295 nm corresponding to the (220) plane of γ-Fe_2_O_3_. The blurry section between TiO_2_ and γ-Fe_2_O_3_ could be attributed to the amorphous PANI. Fig. S2† shows the scanning electron microscopy (SEM) images of γ-Fe_2_O_3_@PANI@TiO_2_. In addition, the morphologies of γ-Fe_2_O_3_ and γ-Fe_2_O_3_@PANI were also studied by TEM technology. Fig. S3† shows the low magnification TEM images of γ-Fe_2_O_3_, indicating the morphology of nanoparticles with the size in the range of 10–50 nm. Fig. S4† shows the γ-Fe_2_O_3_ surrounded by amorphous PANI, which corresponds to the result in Fig. 3b. All results strongly support the successful synthesis of γ-Fe_2_O_3_@PANI.

Fig. S5a† exhibits the N_2_ adsorption–desorption isotherms of γ-Fe_2_O_3_, γ-Fe_2_O_3_@PANI, and γ-Fe_2_O_3_@PANI@TiO_2_ at 77 K. According to the IUPAC classification, all samples show the characteristics of type IV isotherms with an H3 hysteresis loop. No saturated adsorption platform occurs when the value of *p*/*p*_0_ is high, indicating an irregular pore structure. Fig. S5b† shows that the three samples have a similar nature, mainly distributed at 30–70 nm, and concentrated in the mesopore range. PANI coating has a small effect on the pore volume, while an increased pore volume is obtained after introducing TiO_2_. Table S1† clearly shows that γ-Fe_2_O_3_@PANI@TiO_2_ exhibits a high specific surface area, which could provide more active sites for the adsorption of As(iii).

**Fig. 4:**
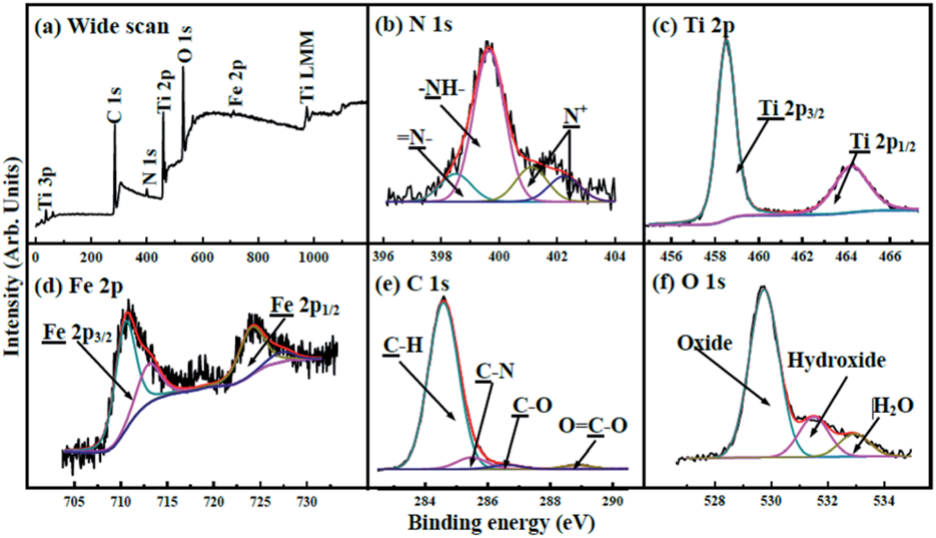
(a) XPS spectrum of γ-Fe_2_O_3_@PANI@TiO_2_. XPS spectra of (b) N 1s, (c) Ti 2p, (d) Fe 2p, (e) C 1s and (f) O 1s regions.

The wide spectrum obtained from X-ray photoelectron spectroscopy (XPS) (Fig. 4a) exhibits that the binding energies (BEs) of C 1s, N 1s, Ti 2p, O 1s, and Fe 2p are 284.3, 400.0, 458.7, 529.9, and 710.6 eV, respectively. The N 1s XPS spectrum (Fig. 4b) shows that amine (–NH–) with BEs at 400.0 eV occupies prominent character in nitrogen atoms.^42^ Imines (=N–) and positively charged nitrogen (N^+^) are also observed. The Ti 2p spectrum (Fig. 4c) exhibits two peaks with BEs at 458.3 and 463.9 eV, corresponding to Ti 2p_3/2_ and Ti 2p_1/2_, respectively.^43^ The Fe 2p XPS spectrum (Fig. 4d) shows two peaks at 710.9 and 724.9 eV that are ascribed to Fe 2p_3/2_ and Fe 2p_1/2_.^41,44,45^ The C 1s spectrum shown in Fig. 4e can be deconvoluted into four components with BEs at about 284.3, 285.1, 286.0 and 288.5 eV, corresponding to C–H, C–N, C–O, and O=C–O, respectively. The O 1s spectrum (Fig. 4f) can be deconvoluted into three peaks; the BEs at 532.9, 531.5, and 529.7 eV can be ascribed to H_2_O (H–O–H), hydroxide (O–H), and oxide (Fe–O and Ti–O), respectively.^42,43^ The XPS patterns show the successful formation of the heterojunction, in good agreement with the results of XRD and FTIR.

Fig. S6 and Table S2† show the magnetization curves and detailed comparison of γ-Fe_2_O_3_@PANI@TiO_2_, γ-Fe_2_O_3_@PANI, and γ-Fe_2_O_3_. A magnetic nuclear shielding effect can be observed after introduction of PANI (43.20 emu g^−1^) and TiO_2_ (18.51 emu g^−1^), while the magnitude of coercive force was not significantly affected, remaining at around 120 Oe. Fig. S7a† shows the UV-vis DRS spectra of all samples. It is noted that the absorption increased from 400 nm for TiO_2_ to 500 nm for γ-Fe_2_O_3_@PANI@TiO_2_, which implies that the absorption of such a heterogeneous junction composite is in the visible light region. Fig. S7b† clearly shows that the γ-Fe_2_O_3_@PANI@TiO_2_ exhibits a narrower forbidden bandwidth (2.49 eV) than TiO_2_ (3.16 eV), indicating transition of more photoelectrons in such a heterogeneous junction composite.

**Fig. 5:**
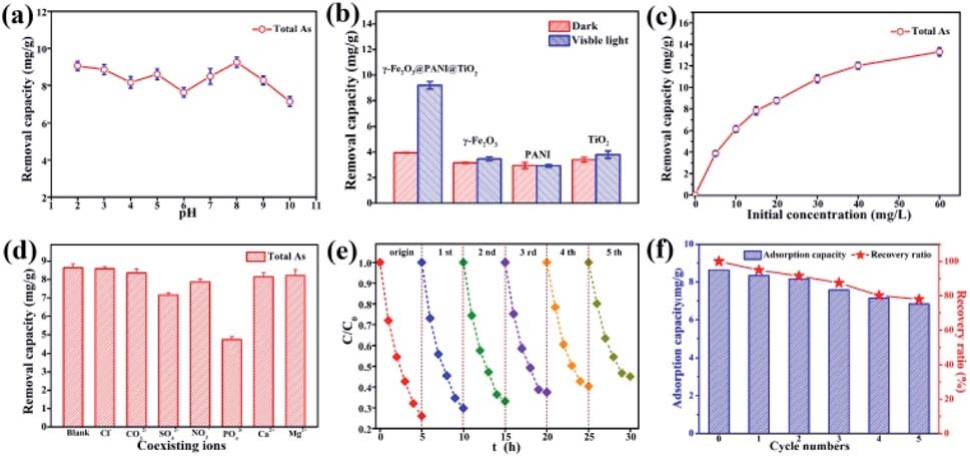
(a) The arsenic removal capacity of γ-Fe_2_O_3_@PANI@TiO_2_ in solutions with various initial pH. (b) The arsenic removal capacity with different adsorbents in the dark and under visible light. (c) The arsenic removal capacity of γ-Fe_2_O_3_@PANI@TiO_2_ at various initial concentrations of As(iii). (d) The effect of co-existing ions on arsenic removal by the γ-Fe_2_O_3_@PANI@TiO_2_. (e) Recycling experiment of the photocatalytic oxidation activity of the γ-Fe_2_O_3_@PANI@TiO_2_ toward As(iii). (f) Recycling experiments of the total As removal by the γ-Fe_2_O_3_@PANI@TiO_2_.

Arsenic removal experiments by photocatalytic oxidation and adsorption are carried out under visible light derived from a Xe lamp with a luminous intensity of 500 W. In the process of photocatalytic oxidation of arsenic, the solution pH will affect the species distribution of arsenic, the surface characteristics of the catalyst, and the position of the energy band, thus affecting the transformation and surface adsorption of As(iii)/As(v). Fig. 5a shows that the total arsenic removal onto the composite material is basically stable (7–9 mg g^−1^) within an initial pH range of 2–10, which is a limitation for using some adsorbents.^46,47^ Based on the pH range of typical arsenic-containing groundwater, a pH of 5.0 was selected to be the initial solution pH in later experiments. Fig. 5b indicates that TiO_2_, PANI, and γ-Fe_2_O_3_ deliver only a low capacity for arsenic removal, but γ-Fe_2_O_3_@PANI@TiO_2_ has an outstanding capacity, implying that such a heterogeneous junction composite is superior to TiO_2_ and γ-Fe_2_O_3_ for arsenic removal.

Fig. 5c shows that As removal increases but gradually levels off with an increase in the initial concentration of As(iii), presumably due to the limitation of active sites. Fig. 5d shows that Cl^−^, Ca^2+^, and Mg^2+^ have almost no negative effect on the As adsorption capacity, while NO_3_^−^ and CO_3_^2−^ have a weak inhibitory effect. However, the corresponding removal capacity of As decreased by 17% in the presence of SO_4_^2−^ and 45% in the presence of PO_4_^3−^, indicating that SO_4_^2−^ and PO_4_^3−^ are greatly competitive. Stability is critical to the catalyst for the photocatalytic oxidation of As. Fig. 5e shows that in the initial and fifth cycles the extent of photocatalytic oxidation of As(iii) can reach 75% and 55%, respectively. Fig. 5f exhibits the As removal capacity of γ-Fe_2_O_3_@PANI@TiO_2_ in successive tests; it still delivers a removal capacity of 6.83 mg g^−1^ and a retention rate of 77.8% after 5 cycling tests. The decreased catalytic activity could be ascribed to the increased recombination of electron–hole pairs, thus reducing the charge separation efficiency. This is further confirmed by photoluminescence measurement (Fig. S8†), which shows that γ-Fe_2_O_3_@PANI@TiO_2_ delivers a more significant fluorescence signal in the visible spectrum after the recycling test.

**Fig. 6:**
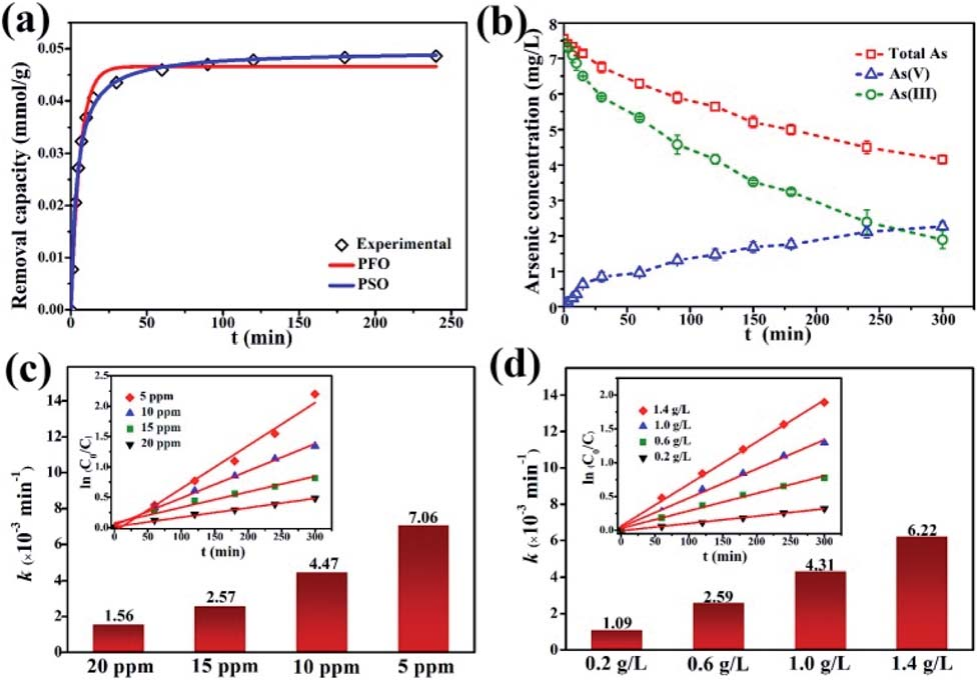
Kinetics analysis of γ-Fe_2_O_3_@PANI@TiO_2_: (a) adsorption kinetics in the dark, (b) reaction kinetics of As(iii) and As(v) under a Xe lamp, (c) the reaction rate constants and fitting curves (inset) at various initial As(iii) concentrations, and (d) the reaction rate constants and fitting curves (inset) at various concentrations of the catalyst.

The kinetics of dark adsorption and light reaction were also investigated. The surface adsorption kinetics and fitting curves of γ-Fe_2_O_3_@PANI@TiO_2_ are shown in Fig. 6a. Within 30 minutes in the dark, the adsorption of As(iii) on γ-Fe_2_O_3_@PANI@TiO_2_ reached up to 90% of the total adsorption quantity, and the rate became slow after 60 minutes with the active hydroxy and amino adsorption sites on the surface being occupied. By fitting the adsorption data with the pseudo-first-order (PFO) and pseudo-second-order (PSO) kinetics model^46,48^ (Table S3†), the PSO model was found to be better with the corresponding non-linear fitting coefficient of Chi-square (*χ*^2^) being as low as 0.970 × 10^−6^ and the corresponding *R*^2^ being 0.996. Therefore, the adsorption process of As(iii) onto γ-Fe_2_O_3_@PANI@TiO_2_ is classified as chemical adsorption. Fig. 6b shows the reaction kinetics of all the arsenic species, including total arsenic, As(iii), and As(v). After dark adsorption, the total arsenic concentration in the solution is 7.5 mg L^−1^. It is noted that As(v) keeps accumulating in the solution and its concentration continually increases, while the total arsenic concentration decreases gradually. After 300 min of light irradiation, the concentration of As(v) in the solution is higher than that of As(iii), accounting for 54% of the total arsenic. The adsorption process of As(iii) is almost balanced, and its photocatalytic oxidation efficiency reaches 75%. The results suggest that the γ-Fe_2_O_3_@PANI@TiO_2_ heterojunction composite is effective for removal of aqueous As(iii) by a coupled photocatalytic oxidation/adsorption process.

The initial concentration of As(iii) is critical to photocatalytic oxidation. The kinetics curves and fitting results of the photocatalytic oxidation of As(iii) at different initial As(iii) concentrations are shown in Fig. S9a† and Fig. 6c. It can be seen that as the initial As(iii) concentration increases, the reaction rate constant decreases. The higher the As(iii) concentration is, the more As(iii) is likely to adsorb onto the catalyst; however, As(iii) itself may also absorb photons, thereby reducing the utilization of light energy by γ-Fe_2_O_3_@PANI@TiO_2_, and thus causing the reaction rate constant to decrease. At the initial As(iii) and catalyst concentration of 5 mg L^−1^ and 1 g L^−1^, the efficiency of As(iii) removal can reach 89%. In addition, Fig. S9b† and Fig. 6d show that γ-Fe_2_O_3_@PANI@TiO_2_ for As(iii) photo-oxidation follows the PFO reaction law of the photocatalytic oxidation process, which is in agreement with earlier reports.^49,50^ Therefore, the reaction process is not only related to the photocatalytic oxidation process, but also affected by adsorption.

**Fig. 7:**
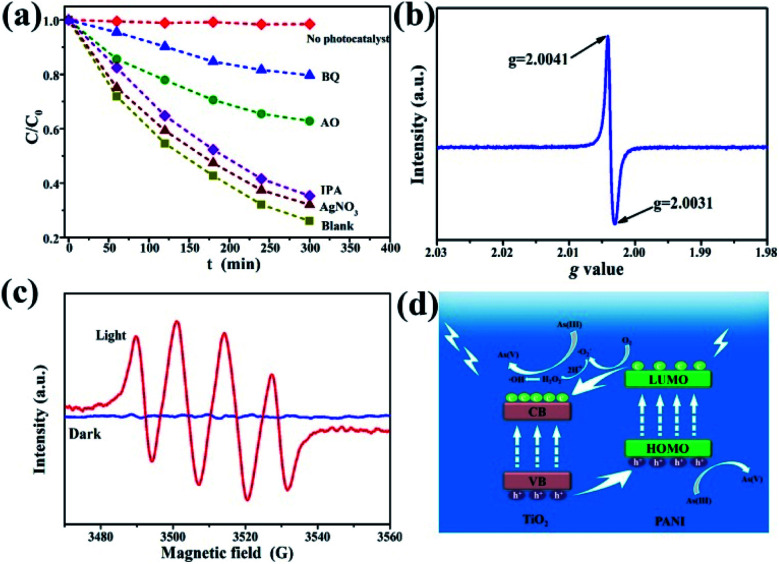
(a) Kinetics of As(iii) photocatalytic oxidation in the presence of different free radical trapping reagents. ESR spectra of the γ-Fe_2_O_3_@PANI@TiO_2_ for (b) light generated holes and (c) DMPO–˙O_2_^−^. (d) Schematic illustration of the postulated mechanism of As(iii) photocatalytic oxidation on the γ-Fe_2_O_3_@PANI@TiO_2_.

To further investigate the possible photocatalytic oxidation mechanism, silver nitrate (AgNO_3_), ammonium oxalate (AO), benzoquinone (BQ), and isopropanol (IPA) were selected as the trapping reagents for electrons, light generated surface holes, superoxide free radicals, and hydroxyl free radicals, respectively.^12^ As(iii) oxidation extent was measured in the presence or absence (blank) of the four reagents. Fig. 7a shows the dynamics curve comparison of photocatalytic oxidation of As(iii) in the presence of the different trapping agents. Almost no oxidation of As(iii) is observed for the case without adding any catalyst. AgNO_3_ and IPA have a weak inhibitory effect on the oxidation of As(iii), indicating that both electrons and hydroxyl radicals have a small effect on the transformation of As(iii) to As(v). With AO as the trapping reagent, the As(iii) oxidation rate is only 36% (*C*/*C*_0_ = 64%) after 5 h of illumination, confirming that photo-produced holes play an important role in the photocatalytic reaction process. For the reaction system with BQ added, the *C*/*C*_0_ was as high as 80%, indicating that the effect of superoxide free radicals was the most prevalent during the As(iii) photocatalytic oxidation. Fig. S10† shows the fitting curves and the corresponding reaction rate constants. Therefore, the four kinds of active substances for the As(iii) photocatalytic oxidation are operative and in the following order: superoxide free radicals > light generated surface holes ≫ hydroxyl radicals and electrons. In this process, however, the oxidation of As(iii) is due to several active substances and their interactions, instead of a single active oxidizer.

In order to further identify the existence of photoproduced holes and superoxide free radicals, electron spin resonance (ESR) technology was used. The ESR spectrum of photogenerated holes (Fig. 7b) shows that the *g* values of the composites are 2.0031 and 2.0041, respectively, and the center of symmetry is 2.0036, corresponding to the Ti^4+^-trapped hole.^51^ Generally the *g* value of TiO_2_ is in the range of 2.0–2.03, and Fe^3+^ can occupy the site of formation of dissolved oxygen free radicals, which affects the *g* value displacement with doped substances to 2.004. Fig. 7c shows the ESR spectra of oxygen-containing free radicals under the conditions of dark and visible light. 5,5-Dimethyl-1-pyrroline *N*-oxide (DMPO) was used as the spin-trapping agent. In the dark, no signal is observed, because TiO_2_ does not have sufficient potential to convert O_2_ to ˙O_2_^−^. However, four response signals related to the DMPO–˙O_2_^−^ are obtained under light irradiation (Fig. 7c).^52^ These results indicate that under visible light irradiation γ-Fe_2_O_3_@PANI@TiO_2_ can produce photoreactive holes and superoxide (hydroperoxy) free radicals, both of which would act as active species in the photocatalytic oxidation of As(iii).

On the basis of the experimental results and other previous studies,^53–55^ we propose the mechanism of photocatalytic oxidation of As(iii) in the visible light system (Fig. 7d). Under visible light irradiation, the low energy level of TiO_2_ with electrons (*i.e.*, VB) absorbs light energy, and the photogenerated holes can transfer to PANI's highest occupied orbital (HOMO) (higher than the valence band of TiO_2_). The lowest empty orbital (LUMO) of PANI is higher than the conduction band of TiO_2_. Therefore, electrons transfer to the TiO_2_ conduction band, which prompts the electrons and holes to move in an opposite direction in the composite catalytic material, reducing the undesirable hole–electron recombination rate, and creating more photogenerated holes. In addition, under weakly acidic conditions, oxygen free radicals (–˙O_2_^−^) and H^+^ can react to generate hydrogen peroxide. The decomposition of unstable hydrogen peroxide continues, thereby generating hydroxide ions, oxygen, and hydroxyl radicals.^56^ Hydroxyl radicals can also participate in the As(iii) oxidation process although with smaller effects (Fig. 7a). During the oxidation of As(iii) to As(v), hydroperoxy free radicals, photogenerated holes, and hydroxyl free radicals play a synergistic role, with the first two being dominant.

## Conclusions

In summary, γ-Fe_2_O_3_@PANI@TiO_2_ has been demonstrated as an effective photocatalytic oxidation adsorbent for As(iii) removal. Compared to its precursors TiO_2_ and γ-Fe_2_O_3_, such a bifunctional material delivers a superior As(iii) removal efficiency under visible light. Notably, it also shows excellent stability at various initial pH values. The investigation of co-existing ions shows that PO_4_^3−^ has the greatest competitive reaction. The photocatalytic oxidation kinetics of As(iii) coincides with the first-order reaction law, which is governed by a coupled photocatalytic oxidation and adsorption. The initial concentration of the catalyst and As(iii) can significantly affect the kinetics of the photocatalytic oxidation; a higher concentration of the catalyst and a lower concentration of As(iii) correspond to a higher rate constant. The photocatalytic oxidation of As(iii) on the magnetic γ-Fe_2_O_3_@PANI@TiO_2_ heterojunction nanocomposite is dominated by the synergy of several active substances with superoxide free radicals and photogenerated holes being the major players. This work provides a new catalyst for As(iii) removal and the associated mechanism of the photocatalytic oxidation of As(iii) is clearly elucidated, which may open up an exciting new window for the rational design and application of heterojunction nanocomposites for photocatalytic oxidation.

## Conflicts of interest

There are no conflicts to declare.

## Supplementary Material

NA-002-D0NA00171F-s001
