# Supplementary material for: A magnetic γ-Fe2O3@PANI@TiO2 core–shell nanocomposite for arsenic removal via a coupled visible-light-induced photocatalytic oxidation–adsorption process
Source: Nanoscale Adv. 2020 Mar 30;2(5):2018–24. doi: 10.1039/d0na00171f (PMC9417276; doi:10.1039/d0na00171f)
Supplement: NA-002-D0NA00171F-s001 [file NA-002-D0NA00171F-s001.pdf]

Electronic Supplementary Material (ESI) for Nanoscale Advances.
This journal is © The Royal Society of Chemistry 2020

Electronic Supplementary Information

Magnetic γ-Fe_2O_3@PANI@TiO_2 Core-Shell Nanocomposites for Arsenic Removal via a Visible-light-induced Photocatalytic Oxidation-Adsorption Coupling Process

Yuan Wang,[a] Ping Zhang,[a] Tian C. Zhang[b], Gang Xiang,[c] Xinlong Wang,[a] Simo Pehkonen,[d] Shaojun Yuan[a,*]

[a] Low-Carbon Technology & Chemical Reaction Engineering Lab, College of Chemical Engineering, Sichuan University, Chengdu 610065, China
[b] Civil Engineering Department, University of Nebraska-Lincoln, Omaha, NE 68182-0178, US
[c] College of Physical Science and Technology, Sichuan University, Chengdu 610064, China
[d] Department of Environmental and Biosciences, University of Eastern Finland, 70211 Kuopio, Finland

*To whom all correspondence should be addressed
Tel: +86-28-85405201, Fax: +86-28-85405201
E-mail: yuanshaojun@gmail.com (S.J. Yuan)

S1 Experimental Section

S1.1 Characterization of the as-synthesized nanocomposites

The Fourier transformed infrared spectroscopy (FTIR Perkin-Elmer L1600300) was carried out to analyze functional groups related information. Raman spectroscopic measurements were performed on a DXR Raman Microscope System (ThermoFisher, USA) with an excitation wavelength of 780 nm. The XRD patterns were obtained on a DX-2007 X X-ray diffractometer (Cu Kα radiation, λ =0.15418 nm, 40 kV, 40 mA). The SEM images of all samples was measured on a JSM-7610F emission scanning electron microscopy (JEOL, Japan). The TEM images were obtained on a Tecnai G2 transmission electron microscopy (JEOL, Japan) at an accelerating voltage of 200 kV. N_2 adsorption-desorption isotherms were recorded by micrometrics instrument (ASAP 2020). X-ray photoelectron spectra (XPS) of the samples were determined by an AXIS ULTRA HAS spectrometer (Kratos Co, UK). Magnetic properties were measured on a vibrating sample magnetometer (East Changing ET9007) with 1 T applied field at an ambient temperature. Thermogravimetric analyses (TGA) was analyzied by a NETZSCHSTA449F3 thermogravimetric Analyzer (NETZSCH, Germany) from zero to 850 °C under nitrogen atmosphere at heating rate of 5 $°C\cdot min^{-1}$. Photoluminescence spectroscopy (FLS920, EDINBURGH INSTRUMENTS) was conducted to analyze the combination of photogenerated holes and electrons with a Xe900 lamp excitation. The optical properties of γ-Fe_2O_3@PANI@TiO_2 nanocomposites were seen in UV-vis DRS analysis (Lambda 750S). The Brunauer-Emmett-Teller (BET) surface area was measured by nitrogen adsorption/desorption isotherms at 77 K using an AutosorbiQ analyzer (Quantachrome, USA). The electron spin resonance (ESR) was conducted with a 300-10/12 electron spin resonance spectrometer (Bruker, Germany) to test the

photogenerated holes and superoxide free radical of the composite materials. Solid powder was directly used in the photogenerated hole experiment. The response signal was the difference between light and dark conditions. The g factor was corrected by standard sample diphenylpyrazine hydrazine (DPPH). The samples were dispersed in 10 mg/L dimethylpyridine n-oxide (DMPO) methanol solution during the test of superoxide free radicals, and the test was conducted after five minutes of visible light irradiation (Xe lamp). The measured magnetic field intensity was 3,400−3,700 G; the microwave frequency was 9.1 GHz, and the microwave intensity was 10 mW.

S1.2 Arsenic removal by photocatalytic oxidation and adsorption experiments

Batch experiments were conducted to examine the performance of magnetic γ-Fe_2O_3@ PANI@TiO_2 heterojunction composites for As(III) photocatalytic oxidation/ adsorption (a coupling process) under the conditions of visible light irradiation, variable initial pH, co-existing ions, etc. The effect of reaction kinetics and associated mechanism were evaluated in different tests (i.e., dark absorption, light reaction, catalyst concentration, initial concentration). Other than specifically identified, all the following tests were conducted in 50-mL test tubes with triplicates (n = 3) in open atmosphere, and the average of the results are reported here.

S1.2.1 Effect of solution pH

The pH value of the solution was adjusted with HCl and NaOH of 0.1 M and 0.5 M, respectively, within a pH range of 2.0−10.0. An aliquot 0.5 g/L of γ-Fe_2O_3@PANI@TiO_2 products were used for the reaction. The initial concentration of As(III) was 20 mg/L.

S1.2.2 Comparison experiments

The pH value of the solution was 5.0. The catalyst dosage was 0.5 g/L, and the

initial concentration of As(III) was 20 mg/L.

S1.2.3 Effect of co-existing ions

To determine the effect of co-existing ion on the As(III) adsorption performance, Cl^- (NaCl), CO_3^{2-} (Na_2CO_3), SO_4^{2-} (Na_2SO_4), NO_3^- ($NaNO_3$), and PO_4^{3-} (H_3PO_4), Ca^{2+} ($CaCl_2$), and Mg^{2+} ($Mg(NO_3)_2$) ions were investigated. The initial concentrations of the ions were 0.267 mmol/L, solution pH of 5.0 and a catalyst dosage of 0.5 g/L.

S1.2.4 Effect of initial As(III) concentration

The initial As(III) concentrations were at 0.067-0.801 mmol/L with initial pH of 5.0 and the catalyst dosage of 0.5 g/L.

S1.2.5 Kinetic experiments

In dark and light irradiation experiments, in the dark adsorption part and the photocatalytic reaction after one, two, three, four, and five hours of irradiation, a sample aliquot was transferred to a colorimetric tube, and making sure the dark sample was not exposed to any light. The pH value of the solution was 5.0, the catalyst dosage was 0.5 g/L, and the initial concentration of As(III) was 10 mg/L. In the experiment of catalyst concentration influence, catalyst amounts of 0.2, 0.6, 1.0, and 1.4 g/L were used. The initial As(III) concentration was set to 5, 10, 15, and 20 mg/L and other reaction conditions were the same.

S1.2.6 Catalyst stability/performance test

Catalyst stability/performance in repeated experiments was also investigated. The initial concentration of As(III) of 20 mg/L and a dose of γ-Fe_2O_3@PANI@TiO_2 of 0.5 g/L were used for 5-h oxidation/adsorption tests; then, the test solution was suspended in 100 ml of 0.5 M NaOH solution, under the conditions of stirring at 100

RPM (to induce desorption) for five hours. After the desorption, the magnets were used for the catalyst separation from water, and the recovered γ-Fe_2O_3@PANI@TiO_2 nanocomposites were washed with a large amount of deionized water, air-dried and then utilized for the next experimental cycle. The above cycle was repeated five times.

S1.2.7 Experiments with a free radical trapping reagent

Free radical trapping agent experiments can determine the role of different typical reactive species in the As(III) photocatalytic oxidation. In this study, four reagents were employed: silver nitrate (6 mmol/L), ammonium oxalate (AO), benzoquinone (BQ) and isopropyl alcohol (IPA) (all at 1 mmol/L) for electrons, photogenerated holes, superoxideradicals and hydroxylradicals, respectively. The pH of the solution was 5.0, the catalyst dosage was 0.5 g/L, and the initial As(III) concentration was 10 mg/L. Controls included test systems with all above ingredients but without 1) adding the trapping reagent (blank), 2) adding the catalyst (no photocatalyst).

S2 Results and Discussion

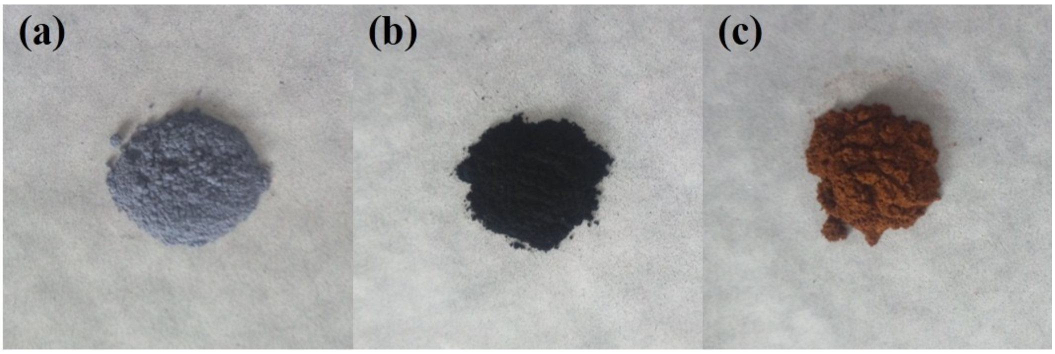

Fig. S1. Optical images of pristine (a) pristine γ-Fe_2O_3@PANI@TiO_2, (b) γ-Fe_2O_3@PANI, and (c) γ-Fe_2O_3 made in this study.

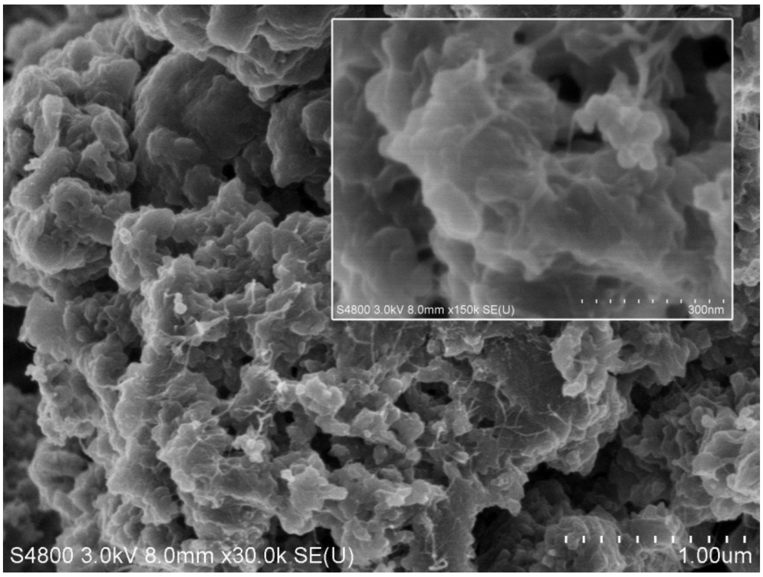

Fig. S2. SEM images of pristine γ-Fe_2O_3@PANI@TiO_2.

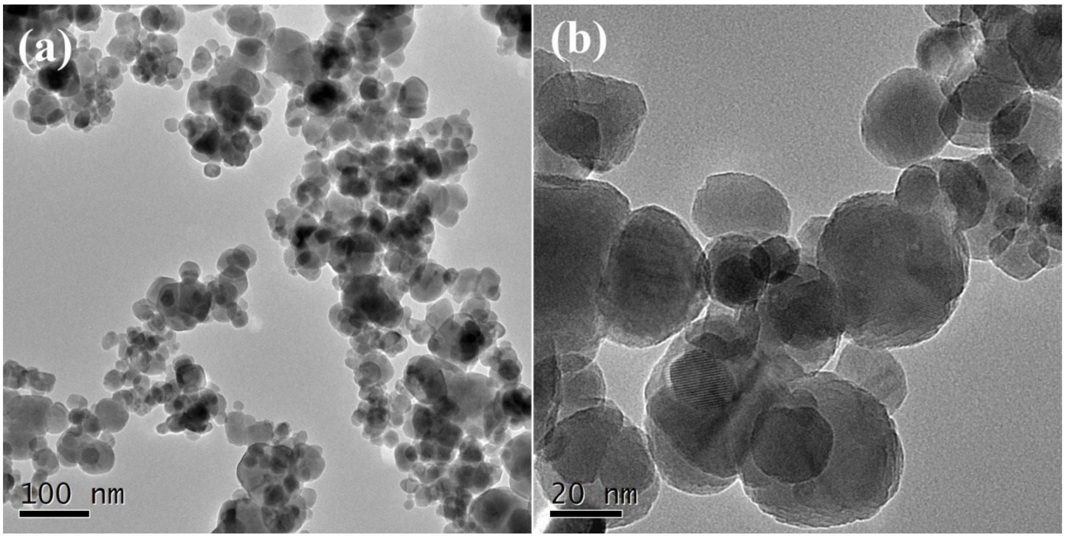

Fig. S3. TEM images (a,b) of the pristine γ-Fe_2O_3 in different magnifications

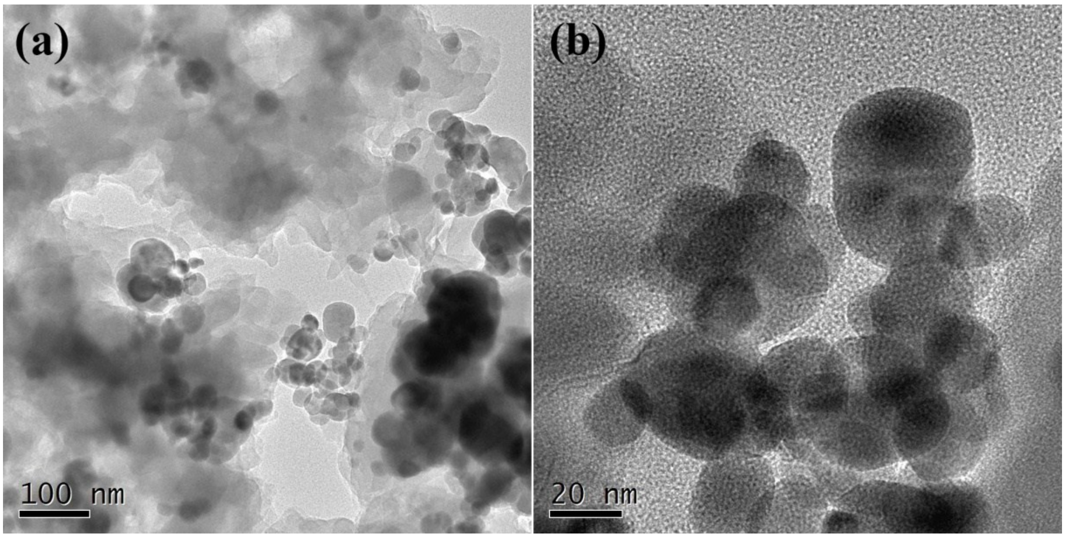

Fig. S4. TEM images (a,b) of the as-synthesized γ-Fe_2O_3@PANI in different magnifications.

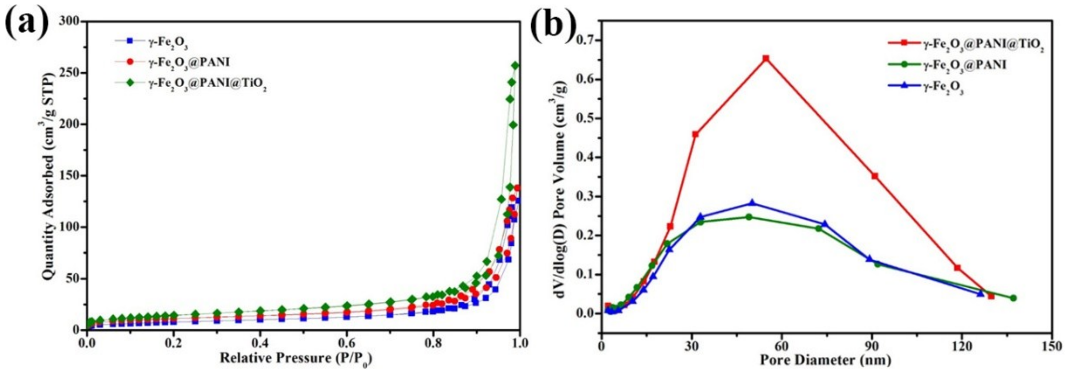

Fig. S5. (a) N_2 adsorption-desorption isotherms at 77 K. (b) Pore size distribution of pristine γ-Fe_2O_3, γ-Fe_2O_3@PANI, and γ-Fe_2O_3@PANI@TiO_2 nanoparticles.

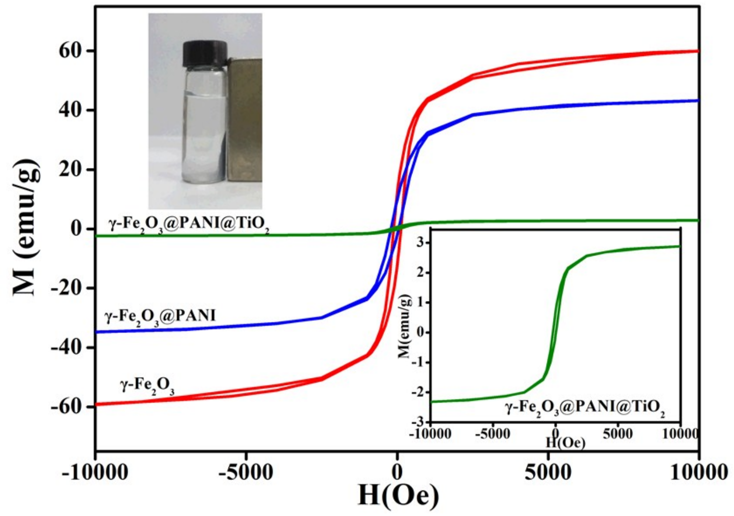

Fig. S6. Magnetization curves of pristine γ-Fe_2O_3@PANI@TiO_2, γ-Fe_2O_3@PANI, and γ-Fe_2O_3. Top insert is photographs of the γ-Fe_2O_3@PANI@TiO_2 suspended in the aqueous solution response to a magnet. Bottom insert is magnetization curve of pristine γ-Fe_2O_3@PANI@TiO_2.

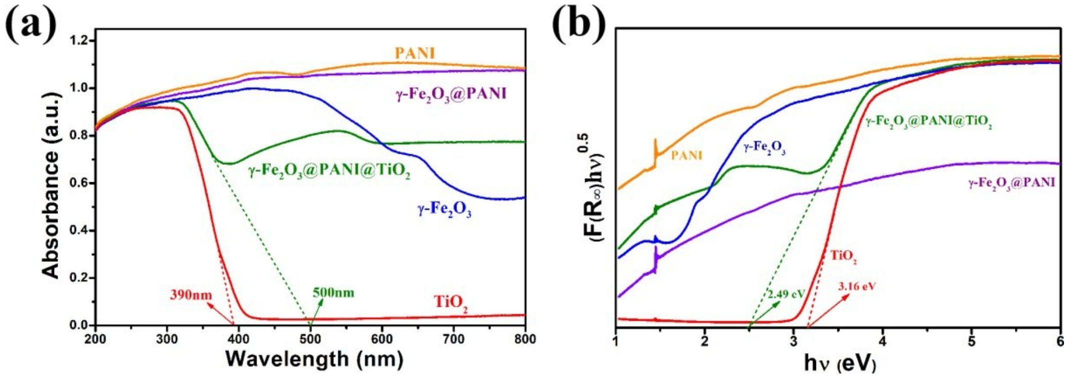

Fig. S7. (a) UV-vis DRS spectra of pristine γ-Fe_2O_3@PANI@TiO_2, γ-Fe_2O_3@PANI, γ-Fe_2O_3, PANI and TiO_2. (b) Computing spectral plot of the corresponding samples.

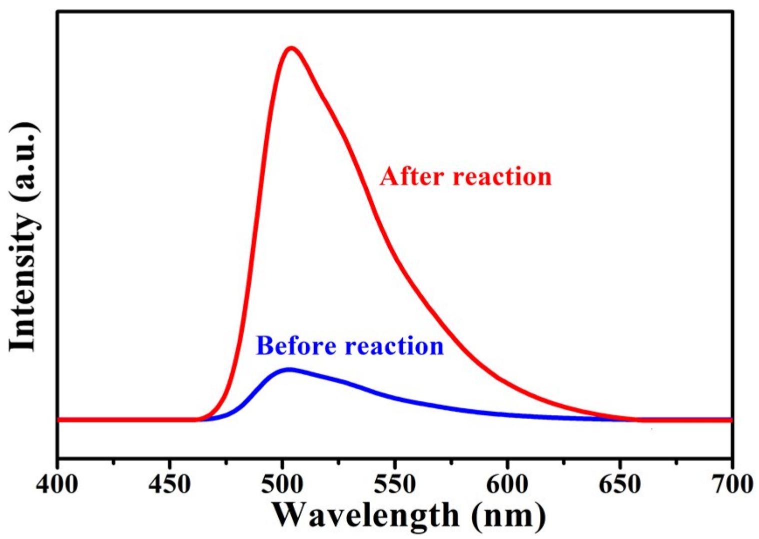

Fig. S8. Photoluminescence spectra of pristine γ-Fe_2O_3@PANI@TiO_2 and that after recycling experiments.

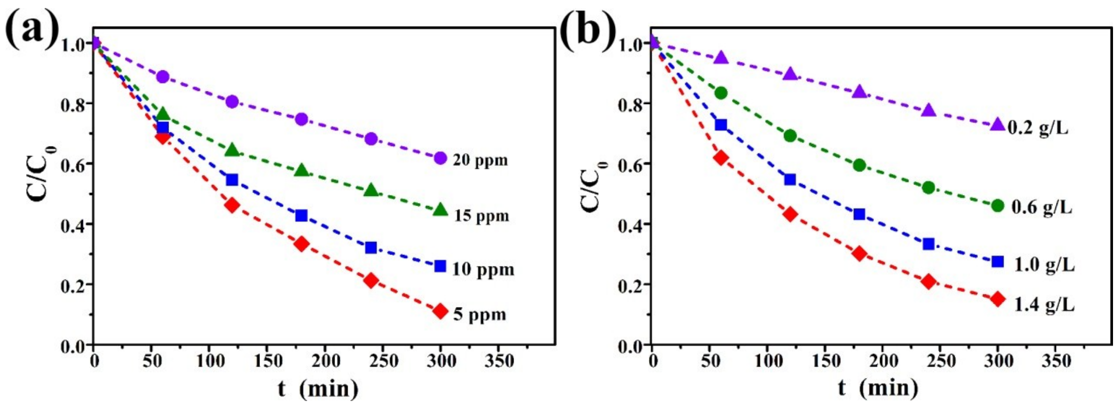

Fig. S9. (a) Kinetics of As(III) photocatalytic oxidation under different initial concentrations of As(III) with pH = 5.0; γ-Fe_2O_3@PANI@TiO_2 dose = 1.0 g/L; t = 5 h. (b) Kinetics of As(III) photocatalytic oxidation under different catalyst concentrations with C_0 of As(III) = 10 mg/L; pH = 5.0; t = 5 h.

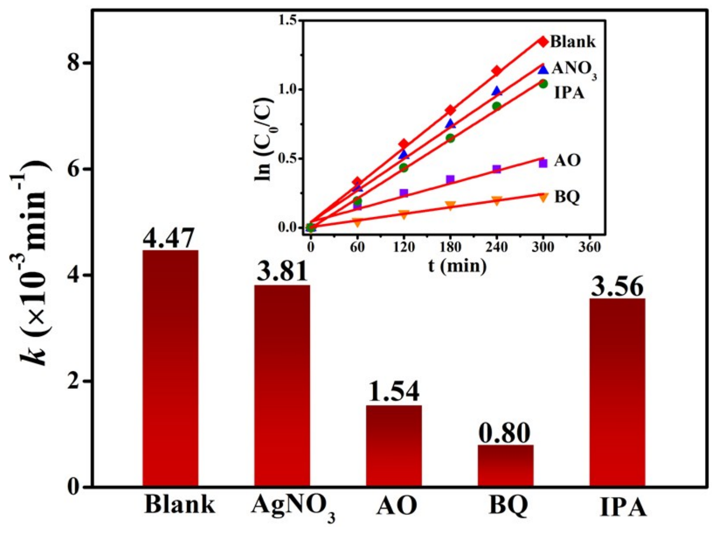

Fig. S10. The results of the fitting curves (inset) and reaction rate constants for different free radical trapping reagents.

Table S1. BET data of γ-Fe_2O_3@PANI@TiO_2.

**Sample name**	**BET Specific surface area (m^2/g)**	**Pore volume (cm^3/g)**	**Average pore diameter (nm)**
**γ-Fe_2O_3**	27.90	0.1893	27.14
**γ-Fe_2O_3@PANI**	39.07	0.2092	25.81
**γ-Fe_2O_3@PANI@TiO_2**	52.03	0.3967	31.79

Table S2. Comparison of magnetic data of three different samples

**Sample**	**Saturation Magnetization (M_s,emu/g)**	**Residual Magnetization (M_r,emu/g)**	**Coercive Force (H_c,Oe)**
**γ-Fe_2O_3**	61.01	13.09	118.84
**γ-Fe_2O_3@PANI**	43.20	5.89	123.92
**γ-Fe_2O_3@PANI@TiO_2**	18.51	0.41	122.87

Table S3. Parameters of kinetic models fitted to the experimental data for the adsorption of As(III) on the γ-Fe_2O_3@PANI@TiO_2 in the dark

Samples	Models	Model parameters				
		k_f (min^{-1})	k_s (g/mmol·min)	q_{max} (mmol/g)	χ^2 (×10^{-6})	R^2
**γ-Fe_2O_3@PANI@TiO_2**	PFO	0.169	−	0.046	2.568	0.990
	PSO	−	5.056	0.050	0.970	0.996

PFO = the pseudo-first-order kinetic model. PSO = the pseudo-second-order kinetic model.
